# TGN-MCDS: A Temporal Graph Network-Based Algorithm for Cluster-Head Optimization in Large-Scale FANETs

**DOI:** 10.3390/s26010347

**Published:** 2026-01-05

**Authors:** Xiangrui Fan, Yuxuan Yang, Shuo Zhang, Wenlong Cai

**Affiliations:** 1 Department of Aerospace Science and Technology, Space Engineering University, Beijing 101400, China; 2 Beijing Aerospace Automatic Control Institute, Beijing 100854, China; 3 Beijing Institute of Technology, Beijing 100081, China; 1120222900@bit.edu.cn

**Keywords:** Flying Ad hoc Networks (FANETs), Temporal Graph Networks (TGN), Minimum Connected Dominating Set (MCDS), cluster-head selection, dynamic network optimization

## Abstract

With the growing deployment of Flying Ad hoc Networks (FANETs) in military and civilian applications, constructing a stable and efficient communication backbone has become a critical challenge. This paper tackles the Cluster Head (CH) optimization problem in large-scale and highly dynamic FANETs by formulating it as a Minimum Connected Dominating Set (MCDS) problem. However, since MCDS is NP-complete on general graphs, existing heuristic and exact algorithms suffer from limited coverage, poor connectivity, and high computational cost. To address these issues, we propose TGN-MCDS, a novel algorithm built upon the Temporal Graph Network (TGN) architecture, which leverages graph neural networks for cluster head selection and efficiently learns time-varying network topologies. The algorithm adopts a multi-objective loss function incorporating coverage, connectivity, size control, centrality, edge penalty, temporal smoothness, and information entropy to guide model training. Simulation results demonstrate that TGN-MCDS rapidly achieves near-optimal CH sets with full node coverage and strong connectivity. Compared with Greedy, Integer Linear Programming (ILP), and Branch-and-Bound (BnB) methods, TGN-MCDS produces fewer and more stable CHs, significantly improving cluster stability while maintaining high computational efficiency for real-time operations in large-scale FANETs.

## 1. Introduction

### 1.1. Challenges in Managing Flying Ad Hoc Networks

Unmanned aerial vehicle (UAV) technology has advanced rapidly in recent years. Operations have evolved from single-drone missions to large-scale cooperative swarms [1]. This evolution has given rise to Flying Ad Hoc Networks (FANETs), which enable real-time coordination and data exchange among UAVs. FANETs are now used in disaster response, environmental monitoring, precision agriculture, border patrol, and temporary communication support [2]. Unlike Mobile Ad Hoc Networks (MANETs) and Vehicular Ad Hoc Networks (VANETs), FANETs exhibit high three-dimensional mobility with six degrees of freedom. Such movement causes frequent and unpredictable topology changes [3]. Most FANETs lack fixed infrastructure and rely on self-organization among nodes. Each UAV also operates under strict limits of energy, computation, and communication range [4]. These characteristics make FANET management highly dynamic and technically challenging.

In large UAV swarms, maintaining a stable and efficient communication backbone is vital for coordinated operation. Cluster-based architectures are widely adopted to organize the network and reduce complexity. They divide the swarm into smaller clusters to improve scalability, energy efficiency, and fault tolerance [5]. Within each cluster, a Cluster Head (CH) manages local nodes, aggregates data, and communicates with other clusters. The CHs and their links form the backbone of the network. The selection of CHs is therefore crucial to system performance. An optimal CH set should provide full coverage, strong connectivity, and minimal control overhead. Achieving this balance under fast-changing topologies remains a difficult multi-objective problem and a core challenge in large-scale FANET management [6,7].

### 1.2. Cluster Head Optimization as a Minimum Connected Dominating Set Problem

The CH selection task in FANETs can be formally modeled as a Minimum Connected Dominating Set (MCDS) problem in graph theory. This formulation captures three essential properties of an optimal CH set: dominance, connectivity, and minimality.

Dominance ensures that every ordinary node is managed or covered by at least one cluster head. Connectivity guarantees that all CHs form a single connected subgraph, supporting global communication across the network. Minimality requires selecting the smallest possible set of CHs while maintaining full coverage and connectivity, which reduces overhead and energy consumption.

However, the MCDS problem [8,9] is NP-hard on general graphs. Exact algorithms are impractical for large and dynamic networks. Traditional heuristic methods, such as greedy algorithms, can find near-optimal solutions in static topologies but fail to adapt to fast-changing structures. In FANETs, a topology that is optimal at one time step can quickly become inefficient or even disconnected due to UAV mobility. To maintain a valid backbone, frequent recomputation of the MCDS is required, leading to heavy computation and control overhead. This instability also causes frequent CH switching, which degrades network performance and reliability.

In addition to heuristic and exact baselines, we further investigated the application of RL/DRL-based decision-making in large-scale FANETs. Existing surveys note that conventional RL is typically suitable for small-scale FANETs, whereas large-scale settings may suffer from enlarged state/action spaces, slow convergence, and the curse of dimensionality; DRL methods also face non-trivial computational complexity and deployment challenges on resource/energy-constrained airborne nodes [10,11]. The study [12] demonstrates that the DRL method achieves average routing times of 20–30 ms and 40–60 ms for 100/300 nodes, respectively. This is difficult to apply to high dynamic flight ad hoc networks.

Therefore, the real challenge in FANETs is not only to find an optimal MCDS at a single moment but to maintain a high-quality and stable MCDS over time. This shifts the problem from static optimization to a dynamic and predictive control task, where adaptability and temporal consistency are crucial for effective network management.

### 1.3. A Learning-Based Paradigm: Temporal Graph Networks

Traditional algorithms for cluster head selection are static and short-sighted. They optimize each network snapshot independently and ignore temporal evolution. Such methods perform poorly when topologies change rapidly.

To overcome this limitation, we introduce a learning-based approach using Temporal Graph Networks (TGNs) [13]. TGNs are designed for dynamic graphs and can capture spatiotemporal dependencies through memory and temporal encoding. By learning how the topology evolves, TGNs produce predictive node representations that support adaptive and stable cluster head selection. This paradigm shifts the problem from static optimization to dynamic learning, enabling proactive decision-making in large-scale FANETs.

### 1.4. Contributions and Paper Organization

The main contributions of this work are summarized as follows.

1. TGN-MCDS Framework.

We propose a Temporal Graph Network-based framework, named TGN-MCDS, for solving the MCDS-based cluster head selection problem in dynamic FANETs. The model integrates a memory mechanism and temporal encoding to learn time-varying network structures in an end-to-end manner. The framework integrates a neighbor discovery module, memory module and time encoding to capture historical topology dynamics—an ability that is absent in many traditional dynamic GNN clustering models.

2. Transform cluster head selection into an equivalent MCDS.

We formulate cluster head selection as the mathematical problem of solving the MCDS, and address it using the TGN-MCDS framework, achieving strong performance. To the best of our knowledge, existing dynamic graph learning methods have not yet been applied to solve the MCDS/cluster head selection problem in large-scale FANETs.

3. Multi-objective Optimization.

We design a composite loss function that jointly optimizes coverage, connectivity, cluster size, centrality, edge penalty, temporal smoothness, and entropy regularization. This design enables the model to balance multiple objectives, improving both connectivity and stability.

4. Efficient unsupervised training and inference.

The model employs an efficient unsupervised training process that leverages memory updates for temporal consistency. During inference, TGN-MCDS produces near-real-time CH predictions suitable for large-scale swarm applications.

5. Performance Evaluation.

Extensive simulations show that TGN-MCDS outperforms classical greedy heuristics, integer linear programming (ILP), and branch-and-bound (BnB) algorithms in both solution quality and stability. The selected CHs are more centralized and less volatile, meeting the demands of dynamic UAV networks.

The rest of this paper is organized as follows.

Section 2 reviews related studies on MCDS optimization and graph neural networks for dynamic graphs. Section 3 presents the system model and problem formulation. Section 4 introduces the proposed TGN-MCDS algorithm in detail. Section 5 discusses the experimental setup and simulation results. Finally, Section 6 concludes the paper and outlines future research directions.

## 2. Related Work

### 2.1. MCDS Optimization and Cluster Head Selection

The Minimum Connected Dominating Set (MCDS) problem is a classical combinatorial optimization problem in graph theory, widely applied to CH selection in wireless ad hoc networks. A connected dominating set provides a virtual backbone that ensures efficient coverage and connectivity among nodes. Guha and Khuller established the computational complexity of the MCDS problem and proposed approximation algorithms with theoretical guarantees [14]. In practical networks, research has focused on distributed heuristic algorithms that construct small connected dominating sets with limited overhead. Wu and Li developed a distributed marking-based algorithm [15], while Wan et al. presented the first message-optimal distributed algorithm that significantly reduces communication costs during CH election [16]. Subsequent studies introduced weighted strategies based on residual energy, node degree, or mobility to enhance CH stability and network lifetime. However, these methods depend on manually designed rules, which restrict adaptability and global optimality in large-scale, dynamic scenarios.

For exact MCDS computation, approaches such as ILP and BnB have been explored. The MCDS can be formulated as a 0–1 integer programming problem and solved using commercial optimization tools; however, the exponential growth of variables limits scalability to small networks. The BnB algorithm improves efficiency through branch pruning based on upper and lower bounds, but its worst-case time complexity remains exponential [17,18]. Although optimized search-based methods have been proposed, they still fail to satisfy real-time requirements in large-scale FANETs. Consequently, practical backbone construction typically relies on heuristic or distributed approximations rather than exact optimization.

### 2.2. Graph Neural Networks and Dynamic Graph Models

With the rise in deep learning, Graph Neural Networks (GNNs) have emerged as powerful models for learning over graph-structured data [19]. GNNs iteratively aggregate neighborhood information to learn node and graph representations that capture both local and global dependencies [20]. Representative variants such as Graph Convolutional Networks (GCNs), GraphSAGE, and Graph Attention Networks (GATs) have achieved remarkable success in applications ranging from social network analysis to communication network optimization [21]. However, these models are designed for static graphs, assuming that node attributes and topologies remain unchanged over time. This assumption limits their applicability to dynamic systems such as UAV networks, where node mobility causes continuous topological variation [22].

To address temporal dynamics, researchers have developed a range of dynamic graph learning frameworks. Early works discretized time into snapshots and applied sequence models such as Recurrent Neural Networks (RNNs) or Long Short-Term Memory (LSTM) networks. Although effective in certain cases, snapshot-based models cannot capture asynchronous or continuous-time events. More recent research directly models continuous-time dynamic graphs (CTDGs), represented by frameworks such as Temporal Graph Attention Networks (TGAT) and Temporal Graph Networks (TGN). TGAT introduces time encoding into the attention mechanism, enabling the learning of time-aware embeddings [23]. Building on this foundation, Rossi et al. proposed the TGN framework, which incorporates node memory modules and flexible message-passing functions to record and update historical states [24,25,26]. TGN updates node memories upon topological events and applies temporal encoding to capture inter-event dependencies [27]. Through the integration of memory and graph operators, TGN achieves superior accuracy and computational efficiency in dynamic representation learning [28,29], outperforming TGAT in tasks such as dynamic link prediction and node classification. This capacity to model continuous and asynchronous structural evolution makes TGN particularly suitable for highly dynamic FANET environments.

Recent research has also applied GNNs to network optimization problems, revealing their potential to learn heuristic strategies for NP-hard graph tasks such as the Maximum Independent Set and Traveling Salesman Problem [30]. These findings indicate that deep learning models can learn efficient combinatorial optimization heuristics directly from data, often outperforming handcrafted methods. Motivated by this paradigm, it becomes natural to explore whether GNNs—particularly temporal variants like TGN—can approximate optimal MCDS-based cluster head selection strategies in dynamic FANETs. By training on diverse and continuously evolving network data, such models can capture implicit spatiotemporal patterns of cluster formation. This paves the way for adaptive, data-driven backbone optimization in large-scale UAV swarms.

## 3. System Model and Problem Formulation

### 3.1. Network Model

A large-scale FANET can be formally modeled as a temporal graph, which represents the dynamic evolution of the UAV swarm topology over discrete time steps. The temporal graph is denoted as $G=\left\{G_{1},G_{2},\ldots ,G_{T}\right\}$, *T* represents the total number of discrete time steps. At any time step t, the network snapshot $G_{t}$ is defined as follows:

- $V\;=\;\left\{v_{1}{,v}_{2}{,\ldots ,v}_{n}\right\}\;$ denotes the fixed set of UAV nodes in the network, where n is the total number of UAVs.
- $E_{t}$ denotes the set of bidirectional communication links at time t. An edge $\left(v_{i\;}{,v}_{j}\right)\;\in {\;E}_{t}$ exists if and only if the Euclidean distance between UAVs $v_{i}$ and $v_{j}$ does not exceed the predefined communication radius R, that is, $distance\left(v_{i},v_{j}\right)\leq R$.
- Each node $v_{i}$ at time t is associated with a feature vector $x_{v}\left(t\right)$, which contains dynamic state attributes such as residual energy ratio, three-dimensional position ${(\mathrm{pos}}_{x}{,\mathrm{pos}}_{y}{,\mathrm{pos}}_{z})$ and velocity vector ${(\mathrm{vel}}_{x}{,\mathrm{vel}}_{y}{,\mathrm{vel}}_{z})$.

This temporal-graph representation effectively captures the essential characteristics of FANETs: while the node set *V* remains relatively stable, both the inter-node connectivity $E_{t}$ and the node states evolve dynamically over time due to UAV mobility and environmental changes.

### 3.2. Problem Definition: MCDS in FANETs

Based on the above network model, the MCDS problem can be defined on each snapshot $G_{t}{\;=\;(V,E}_{t})$. A subset S ⊆ V is a connected dominating set (CDS) of $G_{t}$ if it satisfies the following conditions:

1. Dominating condition:

(1) $$\forall u\;\in \;V\backslash S,\;\exists v\;\in \;S,\;(u,v)\;\in \;E_{t}.$$

This ensures that every non-backbone node $\left(V\backslash S\right)$ is adjacent to at least one backbone node (*S*), thereby guaranteeing full network coverage.

2. Connectivity condition:

The subgraph induced by all nodes in S and the edges among them in $G_{t\;}$, denoted by $G_{t}\left[S\right]$, is connected. This ensures that the backbone forms an integrated and connected structure capable of supporting global information exchange across the network.

3. Minimization objective:

Among all sets satisfying the above conditions, the optimal set $S_{t}^{*}$ has the smallest cardinality:

(2) $$S_{t}^{*}\;=\;\arg\underset{S\subset V}{\min}\left|S\right|,\;s.t.{\;S\;\mathrm{is}\;a\;\mathrm{CDS}\;\mathrm{of}\;G}_{t}.$$

In other words, the MCDS problem seeks to minimize the number of selected nodes while maintaining both coverage and connectivity.

### 3.3. Equivalence to Cluster Head Optimization

The CH selection problem in FANETs can be strictly mapped to the MCDS problem. At any given time t, the optimal cluster head set ${\mathrm{CH}}_{t}$ must constitute a minimum CDS of the network graph $G_{t}$. This equivalence is intuitive and theoretically grounded because it reflects the three fundamental requirements of an ideal CH configuration:

1. Dominating property: Each non-CH node must have a direct communication link to at least one CH node, ensuring complete network coverage and accessibility.
2. Connectivity property: All selected CH nodes must form a connected subgraph, guaranteeing backbone integrity and enabling inter-cluster communication.
3. Minimality objective: The number of CH nodes should be minimized while satisfying the above two properties. This reduces routing overhead, control signaling, and energy consumption while improving management efficiency.

However, solving the MCDS independently at each time step t is a short-sighted approach that neglects temporal stability. Due to frequent topology changes, CH sets optimized at consecutive time steps may differ significantly, resulting in high switching frequency and increased control cost. To address this issue, a more realistic dynamic objective should minimize not only the instantaneous backbone size but also the variation between consecutive time steps. This can be expressed as *a* weighted optimization problem:

(3) $$\min\left[\alpha \cdot {|\mathrm{CH}}_{t}\left|+(1\;-\;\alpha \right)\cdot \frac{{|(\mathrm{CH}}_{t}{\backslash \mathrm{CH}}_{t-1})\;\cup {\;(\mathrm{CH}}_{t-1}{\backslash \mathrm{CH}}_{t})|}{{|\mathrm{CH}}_{t}\;\cup {\;\mathrm{CH}}_{t-1}|}\right].$$

The first term aims to achieve instantaneous optimality (i.e., minimal size), while the second term focuses on ensuring temporal stability. α is a weighting factor balancing compactness and stability. Traditional algorithms cannot directly optimize such time-dependent objectives. This limitation motivates the adoption of TGNs, whose memory and temporal encoding mechanisms enable the model to learn implicit dynamic decision strategies influenced by historical states. When selecting CHs, a TGN does not only evaluate each node’s current domination capability but also its temporal stability potential. For instance, a node that remains central in the network topology across multiple time steps may be assigned a higher CH probability, even if its instantaneous coverage is slightly lower. Through this mechanism, TGNs can approximate the above dynamic composite objective, achieving a balance between instantaneous optimality and long-term stability. This leads to an adaptive and temporally consistent CH selection strategy suitable for large-scale and highly dynamic FANET environments.

## 4. TGN-MCDS: A Temporal Graph Network Approach for MCDS Optimization

To solve the MCDS problem in dynamic FANETs, we propose TGN-MCDS, an end-to-end learning framework. The framework employs temporal graph neural networks to capture spatiotemporal patterns. Each node receives a probability score indicating its likelihood of being part of the MCDS. A lightweight decoder then constructs the final backbone solution from these probabilities through an efficient decoding process.

### 4.1. Architecture Overview

The TGN-MCDS framework consists of two main components: a TGN encoder and an MCDS decoder. The encoder receives a temporal graph stream ${{\{(G}_{t}{,X}_{t})\}}_{t=1}^{T}$ as input *T* represents the total number of discrete time steps. At each time step t, it computes a high-dimensional embedding $h_{v}\left(t\right)$ for each node v. This embedding represents the node’s local topology, intrinsic attributes, and dynamic evolution across time.

The decoder takes the node embeddings ${{\{h}_{v}\left(t)\right\}}_{v\in V}$ generated by the encoder and maps them to scalar probabilities $p_{v}\left(t\right)$ through a shallow multi-layer perceptron (MLP). These probabilities are then used by a probability-guided greedy algorithm to construct the MCDS solution. The entire framework is trained end-to-end with a composite loss function, allowing the model to learn the mapping from raw temporal graph data to high-quality MCDS solutions.

### 4.2. Temporal Graph Network Encoder

The encoder of TGN-MCDS is adapted from the original Temporal Graph Network and customized for dynamic FANETs. It includes four core modules: a memory module, a message generation and aggregation module, an embedding module, and a neighbor-finder module.

#### 4.2.1. Memory Module

The memory module maintains a time-varying hidden state for each node, summarizing its interaction history. Similarly to recurrent network states, the memory updates whenever a topological change or neighbor event occurs and decays smoothly during inactivity. Initially, all memory vectors $s_{i}\left(0\right)$ are zero. At each step, the memory is updated by exponential smoothing:

(4) $$s_{i}(t)\;=\;\beta \;s_{i}^{\mathrm{old}}\;+\;(1-\beta )\;{\tan h(\;\bar{m}}_{i}\left(t)\right),$$

where $s_{i}\left(t\right)$ is the updated memory, ${\bar{m}}_{i}\left(t\right)$ is the aggregated message, and β ∈ [0,1] controls memory retention. This mechanism preserves long-term dependencies while preventing outdated information from dominating. When a UAV node reappears after inactivity, its refreshed memory promptly reflects recent environmental changes, maintaining adaptive behavior.

#### 4.2.2. Message Generation and Aggregation Module

This module defines how node interactions generate messages and how they are aggregated for memory updates. When node  i  interacts with neighbor  j  at time t  (when the distance between them becomes smaller than R), each node produces a message for itself and the neighbor. All adjacency relations within a discrete step are treated as concurrent events. The message from  j  to  i  is computed as:

(5) $$m_{i\leftarrow j}{(t)=x}_{j}{(t)W}_{\mathrm{proj}},$$

where $x_{j}\left(t\right)$ is the neighbor feature of node i, and $W_{\mathrm{proj}}\in R^{D_{\mathrm{in}}{+D}_{t}{\times D}_{\mathrm{mem}}}$ is a fixed projection matrix (non-trainable parameters). Messages from all neighbors are aggregated using mean pooling:

(6) $${\bar{m}}_{i\leftarrow j}(t)\;=\;\frac{1}{{|N}_{i}\left(t)\right|}\sum_{j\in N_{i}\left(t\right)}m_{j}\left(t\right),$$

where $N_{i}\left(t\right)$ denotes the neighbor set of node i.

The resulting ${\bar{m}}_{i}\left(t\right)$ can be regarded as the composite message arriving at node i. To improve efficiency, our implementation simplifies the original TGN design: the message function is replaced with an identity linear transformation on neighbor features, and the aggregator adopts a non-learnable mean function. This simplification maintains model performance while significantly reducing computational complexity, making it suitable for real-time FANET applications.

#### 4.2.3. Embedding Module

The embedding module computes each node’s temporal representation from memory and neighborhood information. It outputs the probability that a node should become a CH. To mitigate memory staleness from long inactivity, embeddings incorporate temporal neighborhood aggregation. At time t, node i builds an input vector ${x_{i}}^{\prime }(t)$ by concatenating three parts: its current feature vector $x_{i}\left(t\right)$, a time-encoding vector $e_{t}$, and updated memory after the previous batch node $s_{i}(t-1)$.

Formally, these are concatenated to form the input embedding:

(7) $${x_{i}}^{\prime }(t)\;=\;\left[x_{i}\left(t\right){,\;e}_{t}{,\;s}_{i}\left(t-1\right)\right].$$

The model uses a two-layer neighbor-aggregation network to produce embeddings. On the first layer, neighbor inputs are averaged, linearly transformed, and passed through ReLU. This yields the intermediate representation $h_{i}^{\left(1\right)}\left(t\right)$. On the second layer, the same aggregation and transformation are applied to $h^{\left(1\right)}$. The second layer output is the final hidden representation $h_{i}^{\left(2\right)}\left(t\right)$.

Layer l is computed as:

(8) $$h_{i}^{\left(l\right)}(t)\;=\;\sigma \left(\frac{1}{\left|N_{i}\right|}\sum_{j\in N_{i}\left(t\right)}h_{j}^{\left(l-1\right)}\left(t\right)W^{\left(l\right)}{+b}^{\left(l\right)}\right),\;l=1,2,$$

where $h_{i}^{\left(0\right)}{(t)=x^{\prime }}_{i}\left(t\right)$ is the input feature vector, with $W^{\left(l\right)}$ and $b^{\left(l\right)}$ as the weight and bias at layer l, and σ as the ReLU activation function.

For a single-layer model, the above operation is performed once. After obtaining the final hidden representation $h_{i}\left(t\right)$ (i.e., $h_{i}^{\left(2\right)}\left(t\right)$ in a two-layer setup), a linear projection followed by a sigmoid activation converts it into the CH probability:

(9) $$z_{i}\left(t\right){\;=\;h}_{i}\left(t\right)W_{\mathrm{out}}{\;+\;b}_{\mathrm{out}},\;p_{i}(t)\;=\;\sigma (z_{i}\left(t)\right).$$

This process effectively combines historical memory and the latest neighborhood information, providing an immediate estimate of each node’s suitability for CH selection.

In the original TGN framework, the embedding module often employs a graph attention mechanism that computes weighted aggregation based on neighbor memories, features, and time encodings. To reduce computational overhead, TGN-MCDS replaces attention with a mean-aggregation graph convolution. Despite this simplification, the inclusion of temporal memory ensures that recent neighbor dynamics are fully captured.

Consequently, the resulting embeddings remain both temporally adaptive and computationally efficient, making the model well-suited for large-scale dynamic FANETs.

#### 4.2.4. Neighbor-Finder Module

The neighbor-finder module determines the set of neighboring nodes used in each graph convolution step. It extracts the spatiotemporal neighborhood of a target node from the dynamic graph sequence.

In the original TGN framework, this module efficiently retrieves historical interaction events from large temporal datasets, often relying on indexing or sampling strategies. In contrast, the neighbor relationships in a FANET are governed directly by spatial proximity. At time t, two UAV nodes are considered neighbors if their Euclidean distance is smaller than the communication range R. Based on this rule, a radius-based adjacency graph G(t) is constructed at each time step using the UAV positions. From G(t), the adjacency matrix A(t) and neighbor list $N_{i}\left(t\right)$ are generated for all nodes. This approach provides a physics-driven implementation of the neighbor-finder: no random sampling or complex indexing is required—neighbor edges are determined solely by spatial distance thresholds.

Because UAV networks exhibit high mobility, the adjacency relations can change at every time step. Through the combination of the memory module and the neighbor-finder, the model simultaneously preserves the influence of past neighbors and incorporates the latest neighborhood information. This design enables TGN-MCDS to capture the impact of dynamic topological evolution on node representations in real time.

#### 4.2.5. Working Mechanism

**Figure 1:**
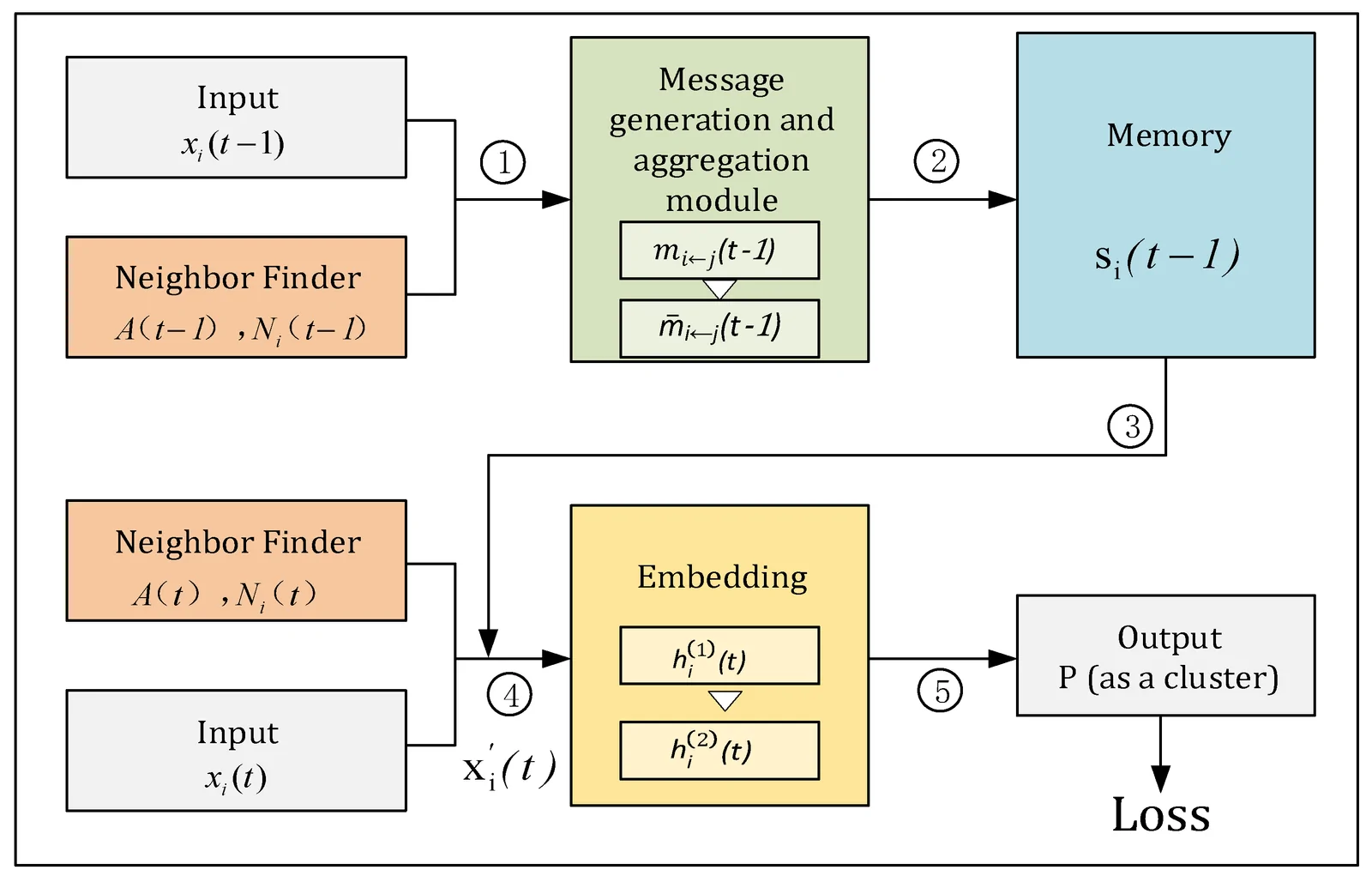
System interaction diagram illustrating the information flow in TGN-MCDS. Here, $x_{i}\left(t\right)$ denotes the current node feature vector, $x_{i}\left(t-1\right)$ is the feature vector at the previous time step, and $s_{i}(t-1)$ is the memory state updated at time *t* − 1. A(t) represents the adjacency matrix at time t, and $N_{i}\left(t\right)$ is denotes the neighbor list (neighbor set) of node *i*. The resulting hidden representation is $h_{i}^{\left(2\right)}\left(t\right)$.

The interactions among all modules in the TGN-MCDS temporal graph encoder are illustrated in Figure 1.

By combining time encoding with memory units, the encoder enables effective information propagation across the dynamic topology of FANETs. The propagation mechanism operates on two complementary dimensions—temporal and topological. Along the temporal dimension, node memories accumulate and decay over time, allowing historical information to flow forward along the time axis. Whenever a node interacts with its neighbors or experiences a neighborhood change, new messages are generated to update its memory. These updated memories continuously influence subsequent embeddings, transmitting past information into future representations. Meanwhile, memories that are not refreshed gradually decay, ensuring that older events exert weaker influence as time progresses.

Along the topological dimension, the embedding module aggregates information from each node’s neighborhood, enabling state diffusion among connected nodes. For example, if a node remains inactive for an extended period but its neighbors are recently active, the aggregation process still allows the node to receive updated contextual information from its vicinity. This design ensures that information remains synchronized across the network even under highly dynamic topology changes. Through this dual propagation mechanism, TGN-MCDS maintains sensitivity to both temporal evolution and structural variation. As a result, the model can balance historical stability with real-time adaptability, producing accurate and robust predictions for CH selection in dynamic FANET environments.

### 4.3. MCDS Decoder and Solution Construction

After obtaining each node’s embedding $h_{i}\left(t\right)$ from the encoder, the decoding process proceeds in two stages.

#### 4.3.1. Probability Prediction

Each embedding $h_{i}\left(t\right)$ is passed through a shallow multi-layer perceptron (MLP). The final layer applies a Sigmoid activation to produce a probability $p_{i}\left(t\right)$, representing the confidence that node i belongs to the optimal MCDS at time t.

#### 4.3.2. Greedy Decoding Strategy

Selecting all nodes with probabilities above a fixed threshold does not necessarily yield a valid MCDS, as it may violate dominance, connectivity, or minimality. To address this, we design a probability-guided greedy decoding algorithm, inspired by classical greedy heuristics but driven by the learned probabilities.

1. Initialization:

Initialize the solution set S = ∅ and mark all nodes as uncovered (white).

2. Dominating Set Construction:

While uncovered (white) nodes remain in the network, the algorithm iteratively expands the dominating set as follows. From all nodes not included in S, select the node $v^{*}$ that maximizes a utility function balancing its selection probability and its ability to cover new nodes:

(10) $${\mathrm{utility}(v)\;=\;p}_{v}(t)\;\times \;|\mathrm{number}\;\mathrm{of}\;\mathrm{white}\;\mathrm{neighbors}\;\mathrm{of}\;v|.$$

Add the selected node $v^{*}$ to the solution set S, and mark $v^{*}$ and all of its white neighbors as covered (gray).

3. Connectivity Enforcement:

After the previous steps, the set S forms a dominating set but may consist of several disconnected components. To ensure full connectivity, the algorithm identifies all connected components within $G_{t}\left[S\right]$, where $G_{t}\left[S\right]$ denotes the subgraph induced by node set S in the current network snapshot $G_{t}$. If multiple components are detected, the algorithm iteratively selects bridge nodes from outside S to connect them. Candidate bridge nodes are chosen based on two criteria:

- A high predicted probability $p_{v}\left(t\right)$
- Their position along shortest paths linking distinct components.

Each selected node is added to S until $G_{t}\left[S\right]$ becomes a single connected subgraph. This process guarantees that the final solution satisfies both the domination and connectivity conditions required by the MCDS formulation.

### 4.4. Model Training, Loss Function, and Supervision Design

#### 4.4.1. Training Procedure

**Figure 2:**
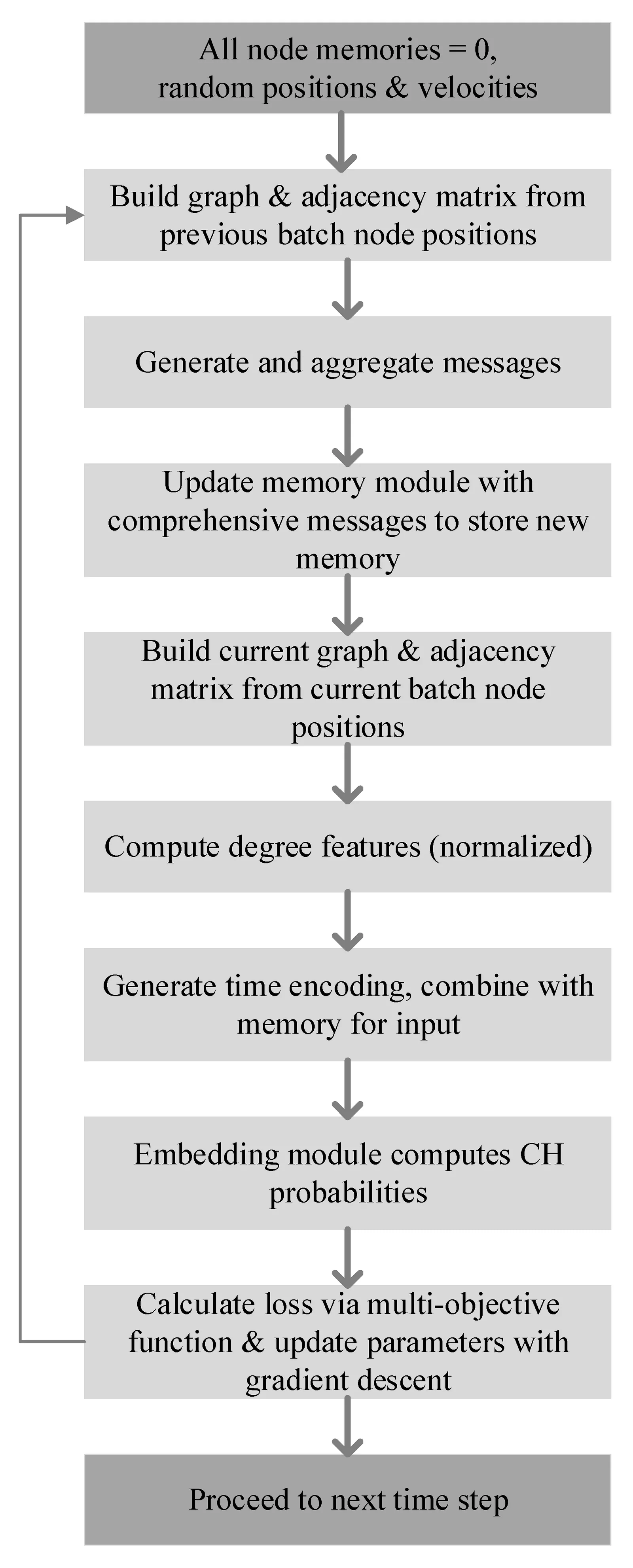
Self-supervised training workflow of the proposed TGN-MCDS algorithm for dynamic cluster head optimization in Flying Ad hoc Networks (FANETs).

The TGN-MCDS model is trained end-to-end in a self-supervised manner using simulated FANET dynamics. Each training epoch simulates the network evolution over T discrete time steps, during which the model continuously observes the changing topology and learns to output the CH probability $p_{i}\left(t\right)$ for every node. At the start of each epoch, all node memories are reset to zero, and the initial node positions and velocities are randomly generated. At every time step, the previous network graph G(t−1) is first constructed from the last batch of node positions, and the message generation and aggregation modules are applied to compute aggregated neighbor information. This information is then used to update the memory state $s_{i}(t-1)$, which stores historical interaction patterns. Next, the current graph G(t) is built based on updated positions, and each node’s normalized degree and other topological features are calculated to form $x_{i}\left(t\right)$. A temporal encoding $e_{t}$ is concatenated with these features and combined with the previous memory to obtain the input $x_{i}^{\prime }\left(t\right)$. The model then performs graph convolution and outputs the CH probability $p_{i}\left(t\right)$. A composite loss L(t) is computed according to the objectives defined in Section 4.4.2, and model parameters are updated through backpropagation using the Adam (Adaptive Moment Estimation) optimizer [31]. The process is executed sequentially without access to future topology, ensuring strict temporal causality. After sufficient epochs, the model parameters converge, enabling the learned network to predict CH probabilities that jointly satisfy domination, connectivity, and stability constraints under dynamic FANET topologies, see Figure 2.

#### 4.4.2. Loss Function and Supervision Design

The training objective of TGN-MCDS is defined as a weighted combination of multiple proxy losses that jointly enforce coverage, connectivity, compactness, and temporal stability:

(11) $$L\;=\;\lambda_{c}L_{c}+\lambda_{n}L_{n}+\lambda_{s}L_{s}+\lambda_{\mathrm{temp}}L_{\mathrm{temp}}+\lambda_{\mathrm{cent}}L_{\mathrm{cent}}+\lambda_{\mathrm{edge}}L_{\mathrm{edge}}+\lambda_{\mathrm{ratio}}L_{\mathrm{ratio}}+\lambda_{h}H$$

Since the CH optimization task lacks explicit supervision labels, these components serve as surrogate signals that guide the model toward learning valid MCDS under dynamic FANET topologies. Coverage and connectivity are treated as hard constraints with the highest weights, cluster size and temporal smoothness are moderately weighted for compactness and stability, and regularization terms are lightly weighted to support convergence.

1. Coverage Loss ($L_{c}$)

This term ensures that every node is dominated by at least one CH. For each node, we define the coverage score $s_{i}=\sum_{j\in \left\{i\right\}\cup N_{i}}p_{j}$, representing the probability that the node or any of its neighbors acts as a CH. The loss penalizes insufficient coverage as

(12) $$L_{c}=\frac{1}{N}\sum_{i=1}^{N}\max(0.1-s_{i}),$$

encouraging the model to achieve full network domination.

2. Connectivity Loss ($L_{n}$)

To maintain a connected backbone among CHs, we compute each node’s neighbor CH probability $r_{i}\;=\sum_{j\in N_{i}}p_{j}$. The connectivity loss is

(13) $$L_{n}=\frac{1}{N}\sum_{i=1}^{N}p_{i}\max({0.1-r}_{i}),$$

which penalizes CHs that lack adjacent CHs, thus promoting topological coherence.

3. Size Loss ($L_{s}$) and Ratio Constraint ($L_{\mathrm{ratio}}$)

The size term

(14) $$L_{s}=\frac{1}{N}\sum_{i}p_{i},$$

controls the average CH ratio, while the ratio constraint

(15) $$L_{\mathrm{ratio}}=(\mu -r_{t}{)}^{2},$$

forces it toward a target proportion $r_{t}$, which gradually decays from 0.30 to 0.15 during training. Together, these two terms ensure compact CH selection and stable convergence.

4. Temporal Smoothness Loss ($L_{\mathrm{temp}}$)

To enhance temporal consistency, the following loss penalizes sharp probability fluctuations between consecutive time steps:

(16) $$L_{\mathrm{temp}}\;=\;\frac{1}{N}\sum_{i}{{(p}_{i}\left(t\right)-p_{i}(t-1))}^{2}.$$

This stabilizes CH selection and mitigates frequent reconfiguration.

5. Centrality and Edge Regularization

To prefer nodes in structurally advantageous positions, two regularization terms are introduced.

(17) $$L_{\mathrm{cent}}=-\frac{1}{N}\sum_{i}p_{i}w_{i}^{\left(\mathrm{cent}\right)}{,\;L}_{\mathrm{edge}\;}=\frac{1}{N}\sum_{i}p_{i}w_{i}^{\left(\mathrm{edge}\right)}.$$

The centrality reward $L_{\mathrm{cent}}$ encourages nodes with higher graph centrality, while the edge penalty $L_{\mathrm{edge}}$ suppresses selections near network boundaries. Weighted by $\lambda_{\mathrm{cent}}$ and $\lambda_{\mathrm{edge}}$, these terms bias CH selection toward central, stable regions.

6. Entropy Regularization (H)

Finally, entropy regularization prevents probability collapse and maintains diversity:

(18) $$H\;=\;-\frac{1}{N}\Sigma_{i}{[p}_{i}{\log\;p}_{i}\;+\;(1-p_{i})\log\;(1-p_{i})].$$

It encourages moderate uncertainty, helping the model explore diverse CH configurations during early training.

All model parameters—including GNN weights, temporal encoders, and memory modules—are optimized using the Adam optimizer. At the end of each epoch, the model is evaluated on randomly generated validation scenarios, measuring coverage rate, connectivity, CH ratio, and total loss. Over time, the losses converge smoothly, and the model learns to generate CH probabilities that satisfy both domination and connectivity constraints under dynamic network conditions. The trained model can then be deployed for online inference, outputting CH probabilities in real time and producing explicit CH sets through the decoding strategy described earlier.

## 5. Experimental Evaluation

This section presents the experimental evaluation of the proposed TGN-MCDS algorithm. We first describe the experimental setup and parameter configuration, followed by comparative analyses of training behavior, coverage and connectivity, CH set size, and computational efficiency. Visualization results are also provided to illustrate the algorithm’s dynamic behavior and overall performance trends.

### 5.1. Experimental Platform and Settings

All experiments were implemented on the MATLAB platform. The hardware environment consisted of an Intel i7 CPU and 32 GB RAM. The simulation was conducted using a three-dimensional mobile network model. We have carried out experiments based on the parameters of node speed, communication distance and coverage range of different network scales. Next, we will introduce the simulation results based on a set of typical experimental parameters, see Table 1.

Each UAV was assigned a communication radius of *R* = 500 m. Two nodes were considered connected if their Euclidean distance was less than *R*, forming an undirected communication link. The network evolution was simulated for *T* = 100 discrete time steps, each corresponding to one second of real time. At every time step, the current network topology  G(t) was reconstructed based on node positions and then fed into the TGN-MCDS model for processing. This setup effectively samples the connectivity dynamics of a continuously evolving FANET at one-second intervals.

### 5.2. Model Configuration and Training Hyperparameters

The graph neural network component of TGN-MCDS consists of two layers. Each node input is represented by its degree feature, while both the hidden and embedding dimensions are set to 32. The network outputs the CH selection probability for each node. The memory vector dimension is also fixed at 32, consistent with the node embedding size. Temporal encoding is configurable but, in the current setup, no explicit time-frequency encoding is applied.

Training is conducted in a self-supervised manner using simulated network data. The composite loss function integrates multiple objectives with the following weighting coefficients: coverage $\lambda_{c}\;=\;4.0$, connectivity $\lambda_{n}\;=\;1.0$, size constraint $\lambda_{s}\;=\;0.03$ (encouraging smaller ∣S∣), and temporal smoothness $\lambda_{temp}\;=\;0.5$. Two structural priors are also incorporated: centrality encouragement $\lambda_{\mathrm{cent}}\;=\;0.10$ and edge penalty $\lambda_{\mathrm{edge}}\;=\;0.12$. An entropy regularization term $\lambda_{h}\;=\;0.01$ is added to enhance training stability. Model optimization is performed using the Adam optimizer with an initial learning rate of ${5\;\times \;10}^{-3}$. Training proceeds for 120 epochs, and every ten epochs the model is evaluated on a validation set to assess coverage rate and CH count, based on which model selection and hyperparameter adjustments are conducted.

### 5.3. Performance Evaluation

To comprehensively evaluate the proposed algorithm, we define several key performance metrics. These indicators not only measure the quality of combinatorial optimization but also directly reflect the operational efficiency of FANETs in real-world scenarios.

#### 5.3.1. Training Convergence

**Figure 3:**
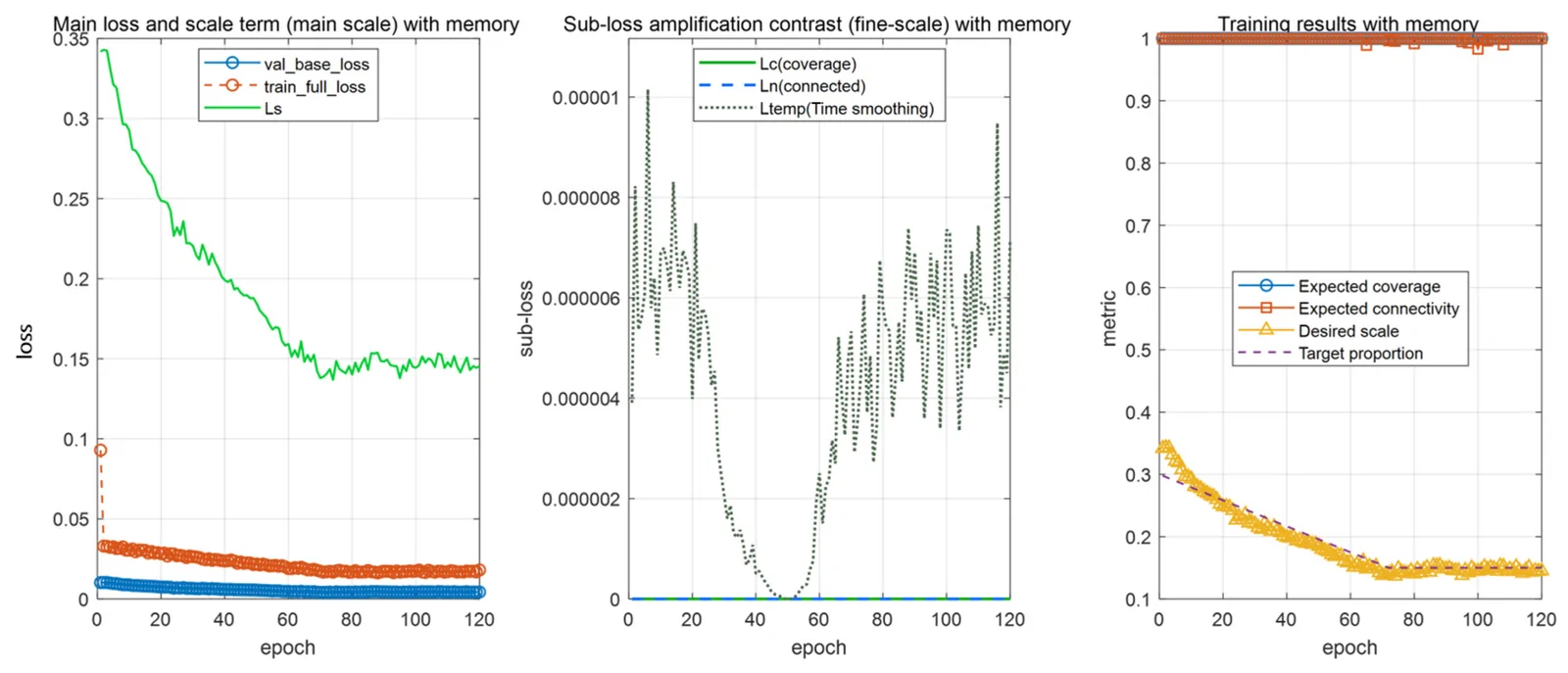
Training loss convergence of the proposed TGN-MCDS model across epochs.

**Figure 4:**
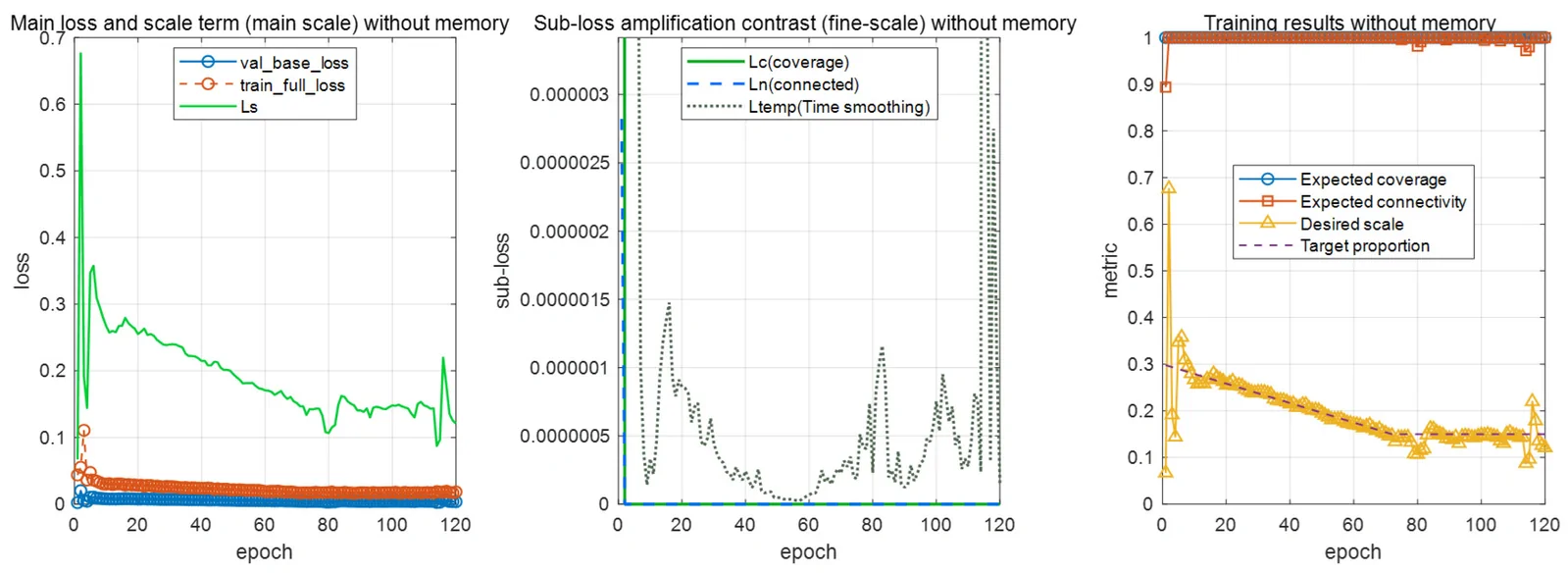
Training loss convergence of the non-memory baseline model.

Figure 3 and Figure 4 illustrate the loss convergence of the TGN-MCDS model and a baseline model without memory modules. The total loss of TGN-MCDS decreases rapidly during early training and stabilizes after approximately 30 epochs. Both the coverage and connectivity losses approach zero within the first few iterations, indicating that the model quickly learns to satisfy basic coverage and connectivity constraints. In later stages, optimization focuses on minimizing the size and entropy losses, refining the number of CHs and improving decision confidence.

As training progresses, the CH ratio gradually decreases from around 0.35 to the target value of approximately 0.15. Throughout the process, both the coverage rate and subgraph connectivity remain close to 1.0. The temporal smoothness loss stays low, suggesting that the CH probabilities evolve smoothly without abrupt oscillations. Compared with the non-memory baseline, TGN-MCDS converges faster and remains more stable. This result confirms that the memory mechanism effectively captures dynamic temporal patterns.

#### 5.3.2. Coverage and Connectivity

We evaluate whether the CH sets selected by TGN-MCDS can fully cover all nodes and form a connected backbone across the test network sequences. As shown in Figure 3, TGN-MCDS consistently achieves nearly 100% node coverage and maintains complete connectivity at all time steps. In contrast, the Greedy heuristic occasionally exhibits coverage below 100% and fluctuating connectivity due to node mobility. The ILP and BnB algorithms, though effective for small-scale graphs, often fail to converge within the predefined time limit in larger networks.

**Figure 5:**
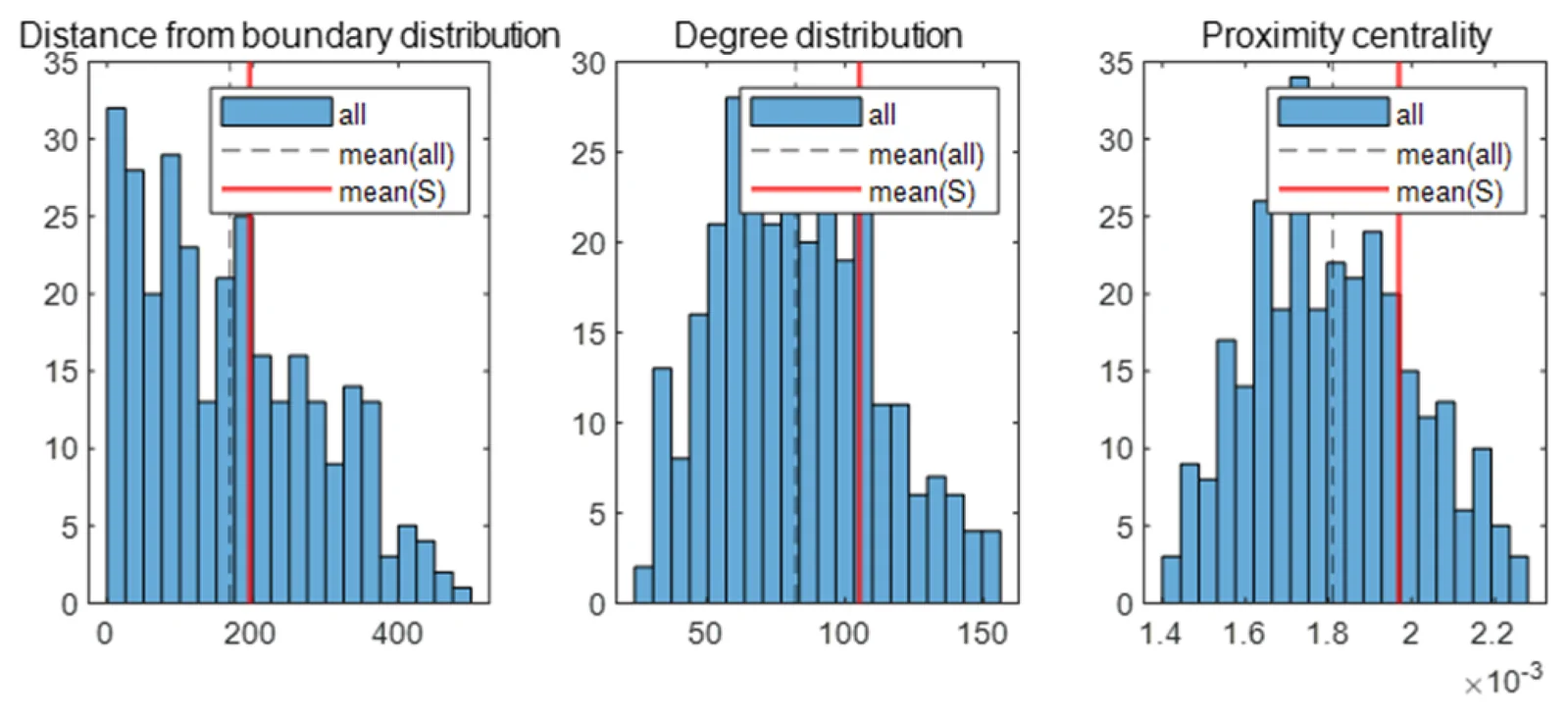
Spatial distribution of the TGN-MCDS solution, including node locations, CH positions, and backbone connectivity.

Guided by the connectivity loss and supported by temporal memory effects, TGN-MCDS strictly ensures both coverage and backbone integrity. Figure 5 further visualizes the spatial distribution of selected CHs, including their proximity to network boundaries, degree distribution, and centrality characteristics.

#### 5.3.3. CH Set Size

**Figure 6:**
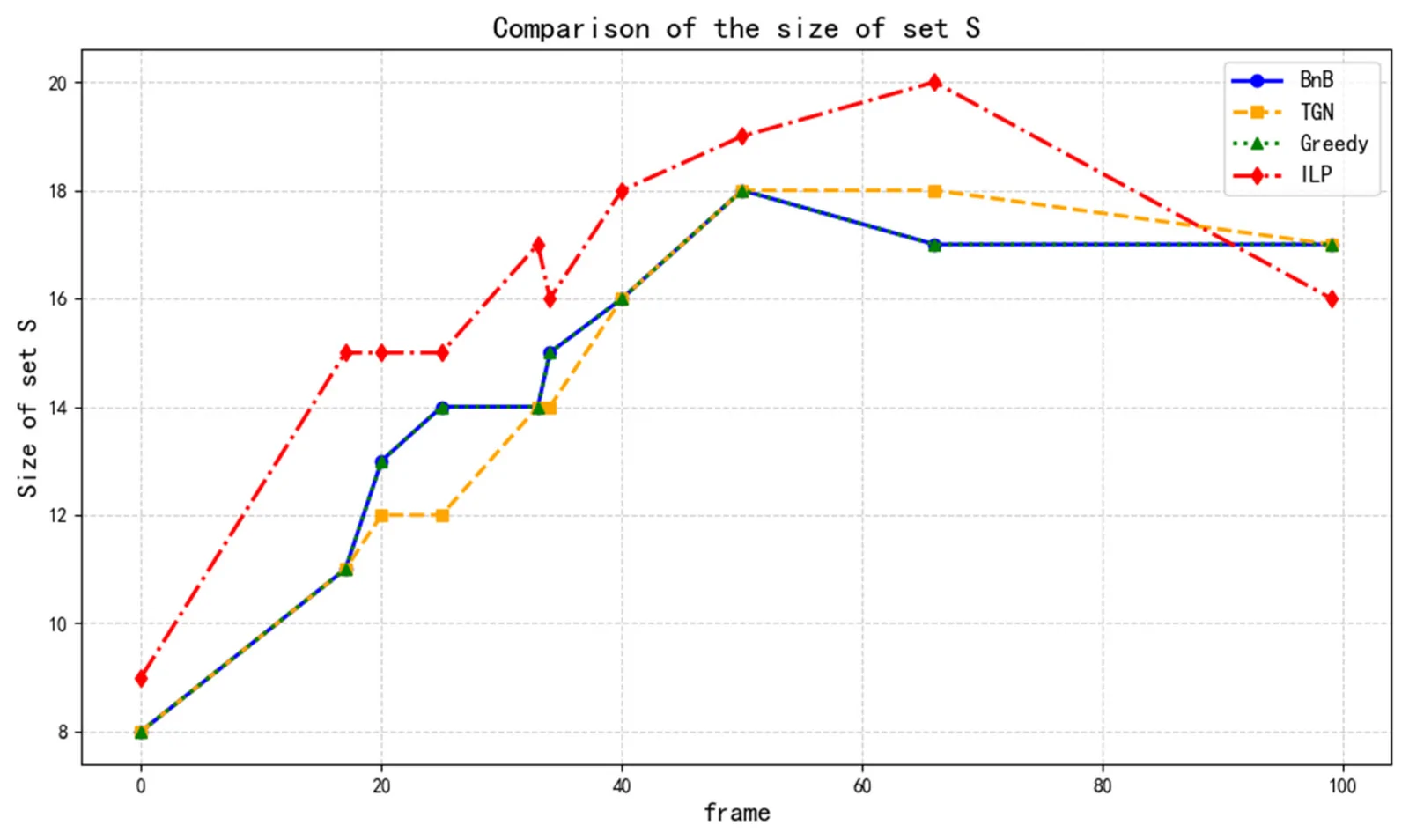
Variation in cluster-head set size ∣S∣ over time under different algorithms.

Figure 6 presents the variation in CH set size ∣S∣ across different time frames. Due to computational limits, ILP and BnB often fail to complete within the maximum allowed runtime. As a result, they are impractical as baselines for large-scale MCDS solutions. Among the four compared methods, TGN-MCDS consistently achieves the smallest CH sets, with only minor deviations at specific frames. This result highlights the model’s strong decision-making capability under complex dynamic topologies.

#### 5.3.4. Cluster Stability

**Figure 7:**
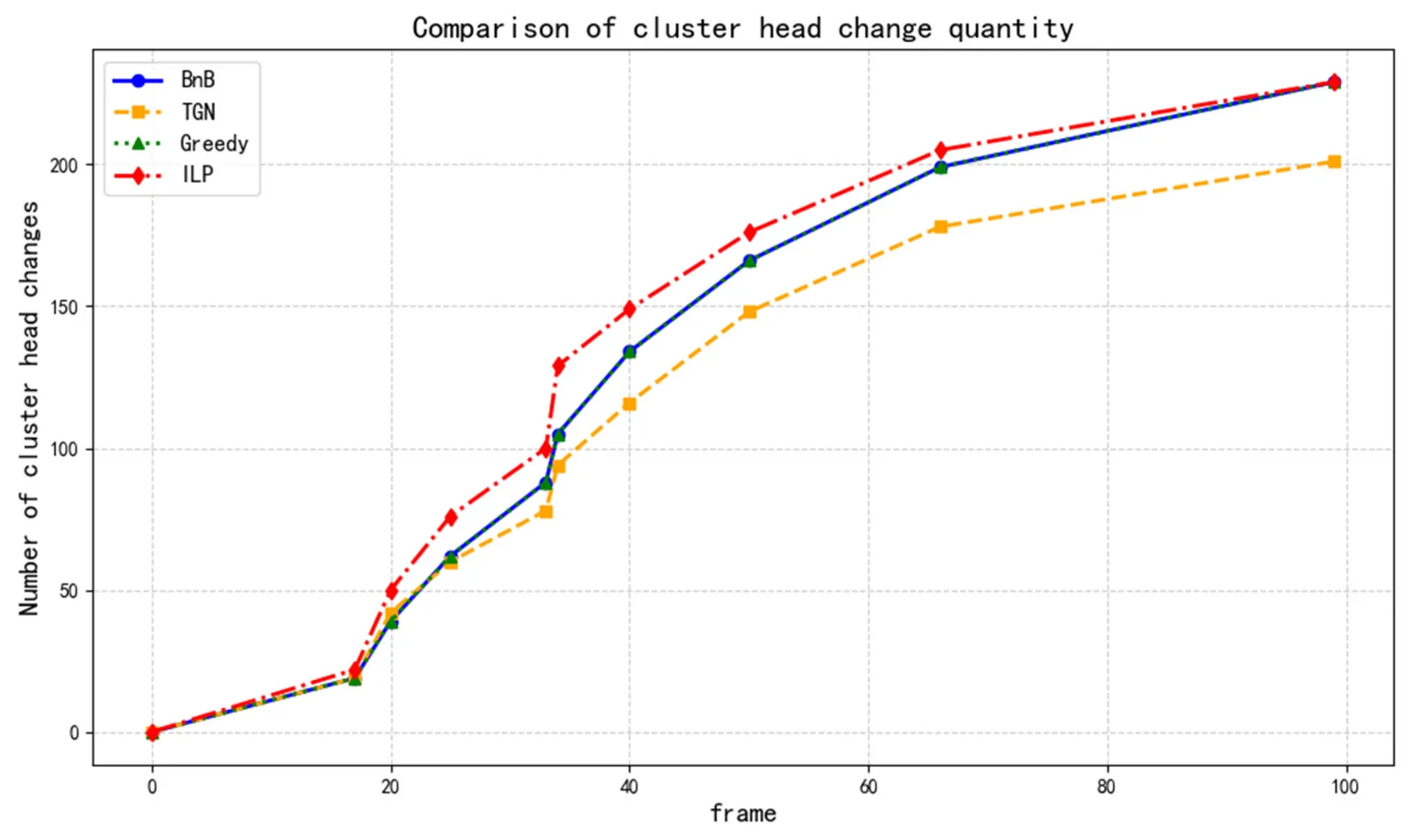
Cluster stability comparison measured by the number of CH transitions across consecutive time frames.

Cluster stability reflects backbone persistence and control overhead. It is evaluated by counting CH changes between consecutive frames and summing them over time. A higher score indicates fewer changes and lower communication cost. In experiments, Figure 7 shows that TGN-MCDS yields far fewer transitions than other algorithms, confirming its strong temporal robustness.

#### 5.3.5. Runtime and Scalability

**Figure 8:**
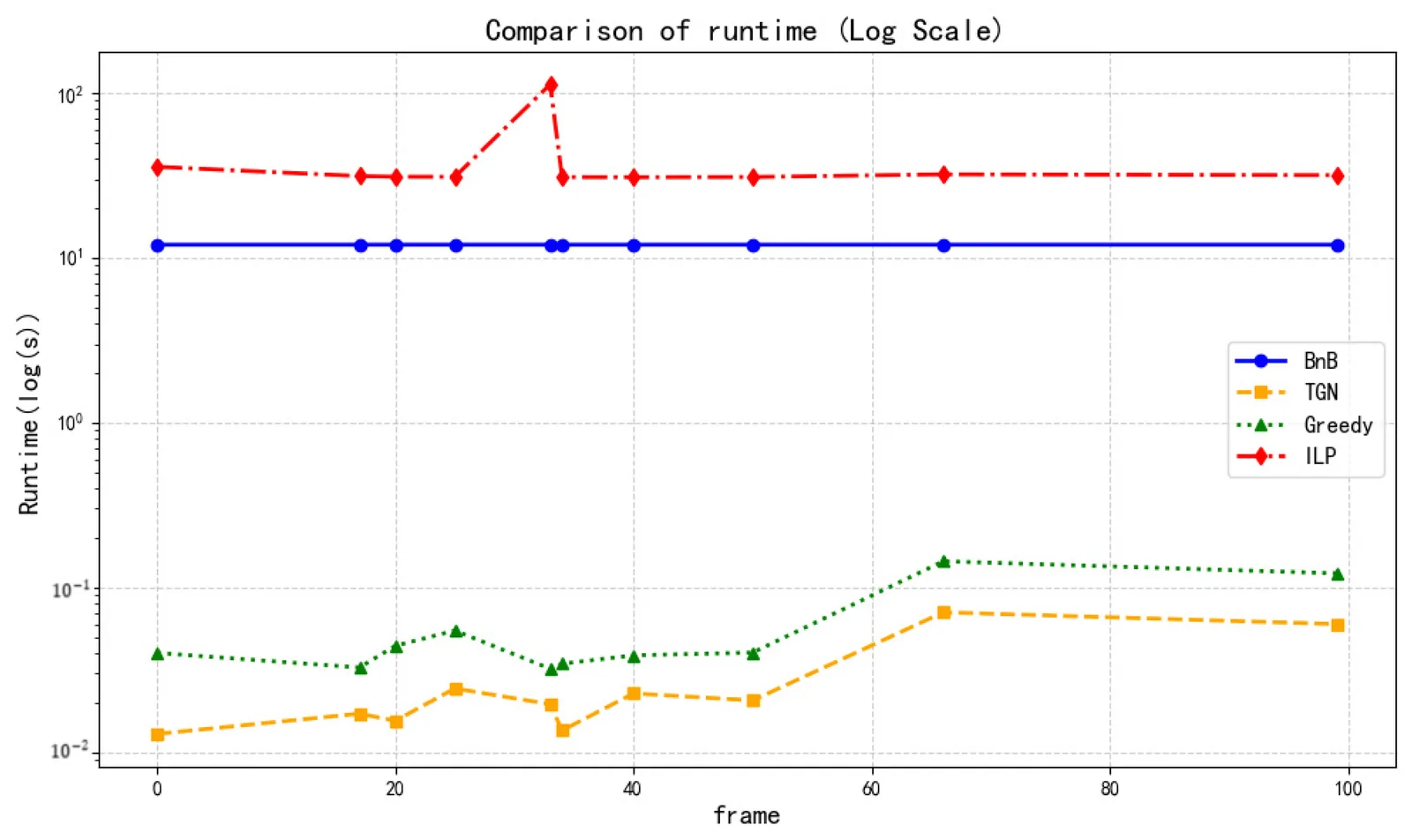
Runtime performance of various algorithms with increasing network size.

We further examine the runtime performance of all algorithms under different network sizes. The ILP method scales poorly: when N > 50, solving time exceeds one minute, and for N > 80, feasible solutions are rarely obtained within an hour. BnB also becomes computationally prohibitive when N ≈ 40. In Figure 8, the latency of BnB and ILP remains nearly unchanged across frames, as the algorithm has reached its maximum runtime limit. The Greedy algorithm has a per-selection complexity of ${O(N}^{2})$, yielding an average runtime of about 0.5 s for N = 1000—acceptable but insufficient for strict real-time demands.

In contrast, TGN-MCDS inference performs only two graph-convolution layers and lightweight memory updates, yielding a linear complexity of $O(\left|E\right|)$. In sparse networks, it achieves high efficiency, requiring only 0.01 s per step for *N* = 100 and 0.05 s for *N* = 1000. With GPU acceleration, the model can further scale to real-time operation in large-scale FANETs. This is well within the capacity of standard embedded processors.

#### 5.3.6. Visualization and Analysis

**Figure 9:**
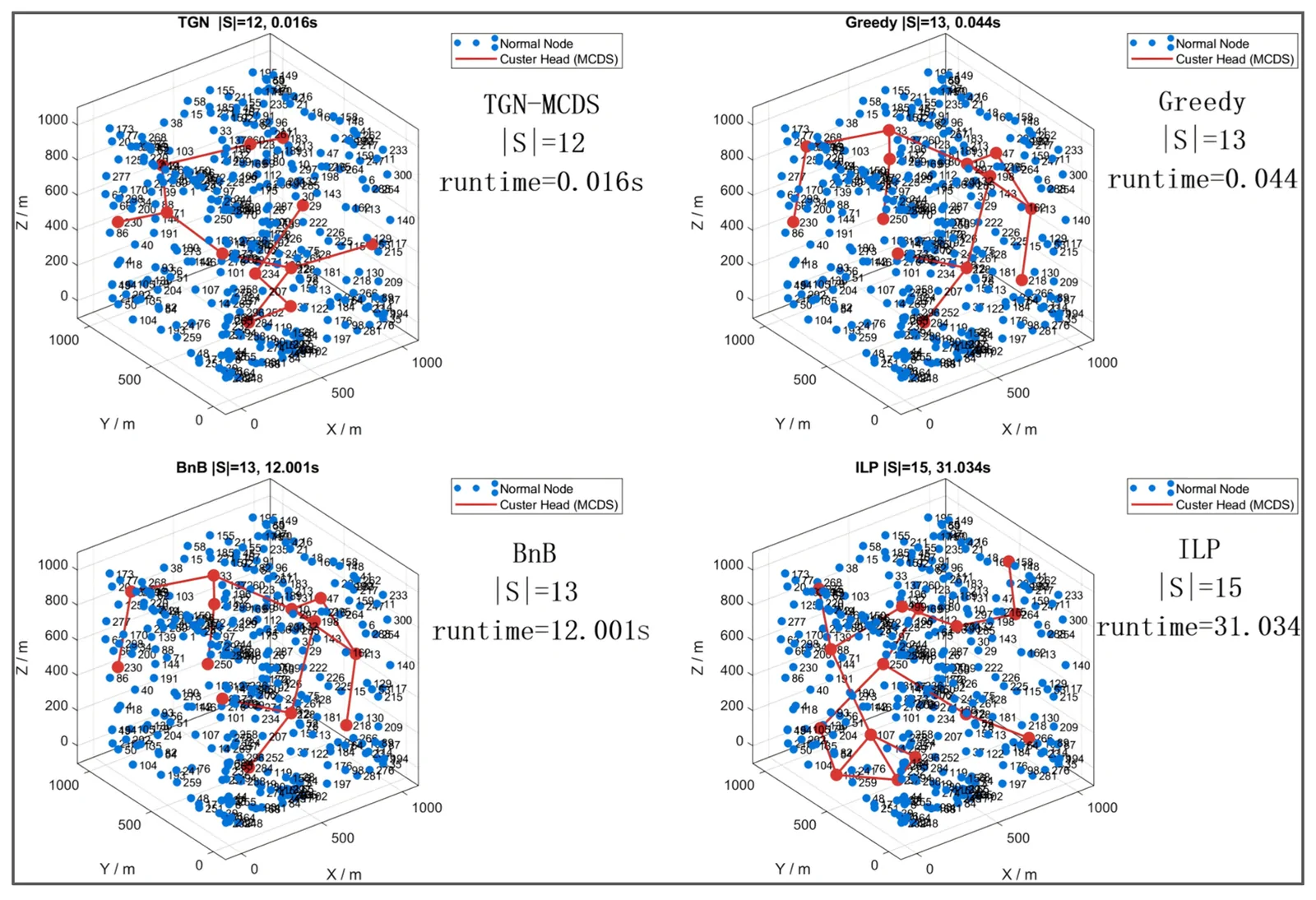
CHs selection visualization of four algorithms at *t* = 20 in a sample FANET topology.

**Figure 10:**
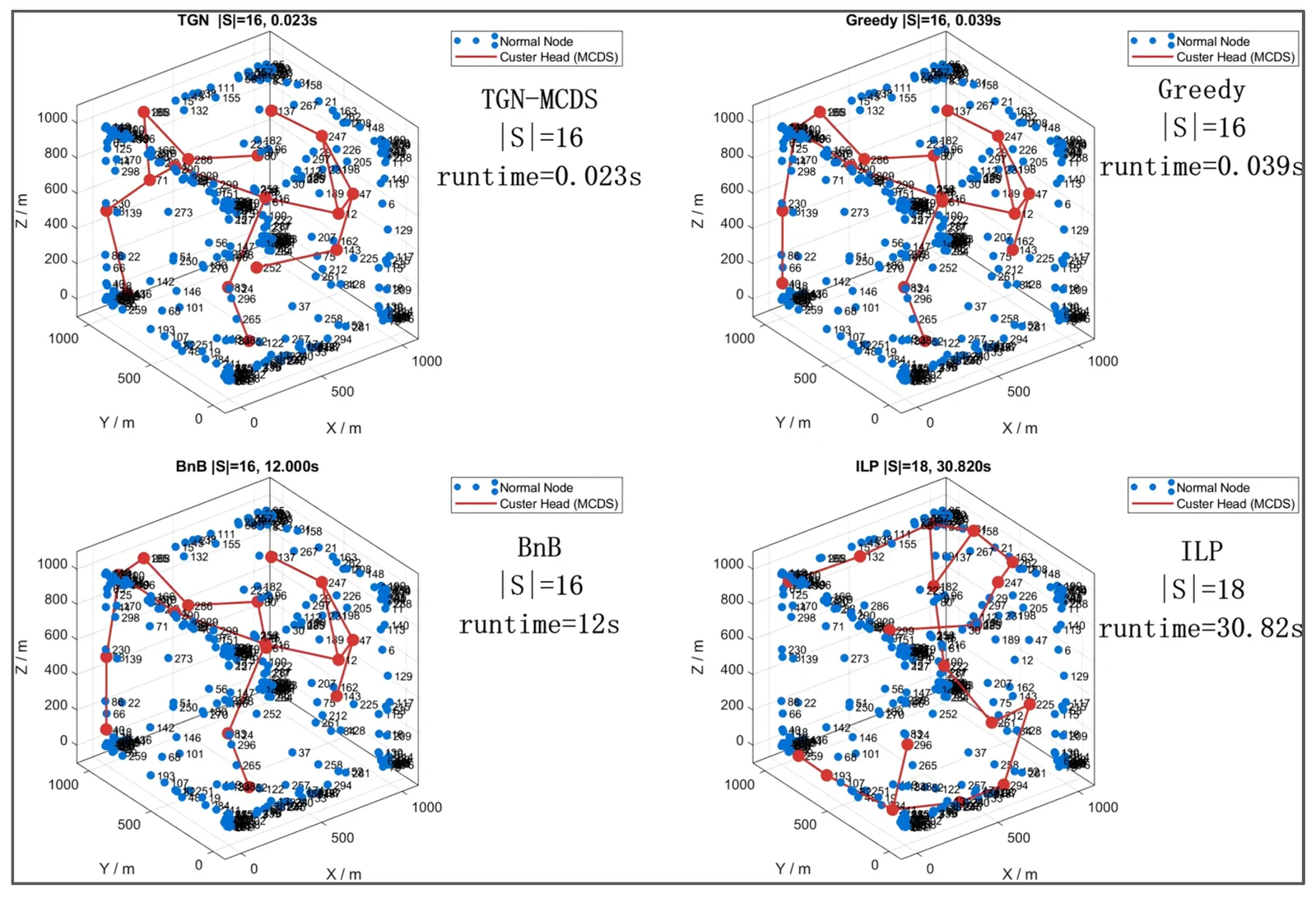
CHs selection visualization of four algorithms at *t* = 50 in a sample FANET topology.

Figure 9 and Figure 10 present a visual comparison of CH selection for a representative FANET topology. At t = 20, the network snapshot shows the node distribution, selected CHs, and backbone communication links. The ILP algorithm produces the largest number of CHs and requires the longest runtime, often reaching the time limit without convergence. Greedy and BnB perform better but still select several nodes near the network boundary. In contrast, TGN-MCDS generates the smallest yet fully connected CH set, covering all critical regions of the network. Most selected CHs are located near the topological center, while peripheral nodes are effectively covered by nearby central CHs rather than being selected themselves. This observation confirms that TGN-MCDS favors central nodes and suppresses redundant selections at the edge of the network.

Over continuous time steps, TGN-MCDS shows smooth transitions in CH evolution. Existing CHs remain active if they still occupy important positions in the updated topology. When a CH moves to an unfavorable region or a better candidate emerges, the model gradually reduces its selection probability and promotes another node. This progressive adjustment prevents abrupt, large-scale replacements and demonstrates high temporal stability. By contrast, the Greedy algorithm recomputes CHs independently at each frame, often causing rapid, unstable changes that appear as “flashing.” This difference highlights the advantages of incorporating temporal memory and smoothness constraints for consistent CH decision-making.

#### 5.3.7. Ablation Experiment

Finally, we conducted a set of ablation studies to evaluate the specific contributions of individual components in the proposed algorithm. The model components are systematically categorized into temporal modules and structural modules, as detailed below.

Memory module: We evaluated a memory-free variant of TGN-MCDS, in which each time step is processed independently without maintaining any historical state. The results indicate a significant degradation in temporal performance: removing the memory module leads to a substantially higher CH switching frequency. In addition, the memory-free model exhibits slightly degraded coverage consistency. In some cases, due to the lack of temporal context for anticipating topology evolution, the coverage rate temporarily drops below 100%. Simultaneously, we observed that when the memory vector of a CH node remains stable across consecutive time steps, the node exhibits a strong tendency to persist within the CH set. Conversely, significant fluctuations in a node’s memory—indicative of substantial changes in its local environment—typically precipitate a transition in status, leading the node to either gain or lose its CH role. Indeed, we identified a strong positive correlation between the magnitude of a CH node’s memory update and the probability of it being replaced in the subsequent time step. This demonstrates that the memory module effectively captures temporal patterns to ‘hand over’ the cluster head role at appropriate moments.

Temporal smoothing loss: We further removed the temporal smoothing loss from the training objective. This variant still retains the memory module but no longer explicitly penalizes rapid temporal changes. We observe that, without the smoothing term, the model becomes noticeably less stable, exhibiting a higher CH switching frequency than the full TGN-MCDS. While coverage and connectivity remain high—as expected, since they are enforced by other loss terms—and the model converges to a similar average number of CHs, it achieves these objectives through substantially more oscillatory CH assignments. This confirms that the temporal smoothing loss is effective in suppressing unnecessary reconfigurations and plays a complementary role to the memory module.

Structural prior terms (centrality reward and edge penalty): We also evaluated a variant in which the structural prior terms are removed. Although the resulting model still satisfies coverage and connectivity constraints, we observe subtle yet consistent differences in CH selection. Specifically, without structural priors, the model tends to select UAVs near the topological boundary of the network as CHs, whereas the full model systematically avoids such selections. Consequently, to maintain full coverage, the prior-free variant requires on average approximately 10% more CHs, since boundary CHs cover fewer neighbors and are therefore less efficient. Due to this suboptimal CH placement, the network also exhibits slightly reduced overall efficiency. These results indicate that, although the centrality reward and edge penalty are not strictly necessary for feasibility, they effectively guide the model toward more efficient CH configurations and thus improve solution quality, see Table 2.

The simulation results clearly demonstrate the effectiveness and superiority of TGN-MCDS in solving the MCDS-based CH optimization problem. The proposed method achieves near-complete coverage with a minimal CH set, while maintaining high temporal stability and computational efficiency—capabilities rarely achieved together by traditional approaches. In large-scale and highly dynamic FANETs, TGN-MCDS provides a robust and efficient solution for enhancing self-organization and communication reliability across UAV networks.

## 6. Conclusions

### 6.1. Summary of Contributions

This paper presents TGN-MCDS, a Temporal Graph Network-based framework for solving the MCDS problem in large-scale FANETs. By incorporating time encoding and memory modules into a graph neural network, the proposed model learns adaptive CH selection strategies directly from dynamic topological data. A multi-objective loss function jointly optimizes coverage, connectivity, cluster size, centrality, and temporal smoothness, ensuring both structural efficiency and stability. Simulation results show that TGN-MCDS consistently outperforms classical heuristic and static approaches in coverage, connectivity robustness, CH reduction, and temporal consistency. These improvements make it a strong candidate for practical FANET scenarios such as autonomous coordination, military reconnaissance, and emergency communication.

### 6.2. Key Findings

Extensive experiments conducted in dynamic FANET environments confirm the effectiveness of the proposed framework. While learning-based models generally produce smaller dominating sets than heuristic methods, the true advantage of TGN-MCDS lies in its ability to capture and anticipate network dynamics. The integrated memory mechanism enables the model to retain long-term temporal information, enabling more stable and predictive CH decisions. Consequently, the resulting communication backbone is compact, persistent, and exhibits minimal reconfiguration over time. This stability leads to measurable performance gains, including prolonged network lifetime and improved data delivery reliability. Overall, the findings highlight an essential insight: optimization in dynamic networks must evolve from static, reactive designs to dynamic, predictive strategies that account for temporal behavior.

### 6.3. Future Work

This work demonstrates the potential of dynamic graph neural networks for optimizing highly mobile UAV systems. Future research can further enhance TGN-MCDS in several directions:

1. Scalability.

Extend the model to very large UAV swarms with thousands of nodes. Promising approaches include graph sampling, hierarchical aggregation, and lightweight GNN architectures.

2. Directed-topology modeling.

Adapt the framework to directed communication graphs to better capture link asymmetry in real-world FANET deployments.

3. Onboard implementation.

Deploy trained TGN models on UAV platforms with limited resources, enabling distributed and real-time inference. Model compression methods such as pruning and quantization will be key to this effort.

4. Cross-layer optimization.

Integrate backbone construction with routing protocols to create co-optimized, topology-aware communication strategies.

5. Heterogeneous UAV networks.

Generalize the model to heterogeneous swarms where UAVs differ in communication range, energy capacity, and mobility patterns, enhancing its adaptability to practical missions.

## Figures and Tables

**Table 1:** Simulation Parameters.

Parameter	Value	Parameter	Value
Number of nodes N	300	Maximum hop count hop	2
Mobility model	Gaussian–Markov	Communication radius R (m)	500
$\mathrm{Average}\;\mathrm{speed}\;\bar{v}$ (m/s)	100	Simulation area (m^3^)	1000 × 1000 × 1000
Propagation model	Nakagami	Simulation time T(s)	100

**Table 2:** Ablation Study: Impact of Key Components on Network Performance.

Model Variant	Component Configuration			Performance Metrics		
	Memory	Temp. Smooth	Struct. Prior	Switching Freq.	Coverage Rate	Avg. CH Count
w/o Memory	**×**	✓	✓	↑↑ (Severe)	<100% (Unstable)	--
w/o Smoothness	✓	**×**	✓	↑ (≈1.3×)	100%	≈Baseline
w/o Struct. Priors	✓	✓	**×**	Low	100%	↑ 10% (Inefficient)
TGN-MCDS (Ours)	✓	✓	✓	Lowest	100% (Stable)	Optimal

Note: “✓” and “**×**” denote the inclusion and exclusion of the component, respectively. ”↑” indicates an increase in the metric (negative impact for switching and count). “↑↑” denotes a significant degradation.

## Data Availability

The original contributions presented in this study are included in the article. Further inquiries can be directed to the corresponding author.

## References

[1] Hussain A., Li S., Tariq H., Lin X., Ali F., Ali A. (2024). Computing Challenges of UAV Networks: A Comprehensive Survey. Comput. Mater. Contin..

[2] Chandran I., Vipin K. (2024). Multi-UAV Networks for Disaster Monitoring: Challenges and Opportunities from a Network Perspective. Drone Syst. Appl..

[3] Bekmezci I., Sahingoz O., Temel Ş. (2013). Flying Ad-Hoc Networks (FANETs): A Survey. Ad Hoc Netw..

[4] Zhang P., Chen S., Zheng X., Li P., Wang G., Wang R., Wang J., Tan L. (2025). UAV Communication in Space–Air–Ground Integrated Networks (SAGINs): Technologies, Applications, and Challenges. Drones.

[5] Zhou R., Zhang X., Song D., Qin K., Xu L. (2023). Topology Duration Optimization for UAV Swarm Network under the System Performance Constraint. Appl. Sci..

[6] Ye L., Zhang Y., Li Y., Han S. A Dynamic Cluster Head Selecting Algorithm for UAV Ad Hoc Networks. Proceedings of the 2020 International Wireless Communications and Mobile Computing (IWCMC).

[7] Khan A., Khan S., Fazal A.S., Ullah F., Ayaz M., Javed M.A. (2021). Intelligent Cluster Routing Scheme for Flying Ad Hoc Networks. Sci. China Inf. Sci..

[8] Purohit G.N., Sharma U. (2010). Constructing Minimum Connected Dominating Set: Algorithmic Approach. Int. J. Appl. Graph Theory Wirel. Ad Hoc Netw. Sens. Netw..

[9] Li R., Hu S., Liu H., Li R., Ouyang D., Yin M. (2019). Multi-Start Local Search Algorithm for the Minimum Connected Dominating Set Problems. Mathematics.

[10] Lansky J., Ali S., Rahmani A.M. (2022). Reinforcement learning-based routing protocols in flying ad hoc networks (FANET): A review. Mathematics.

[11] Rezwan S., Choi W. (2021). A survey on applications of reinforcement learning in flying ad-hoc networks. Electronics.

[12] Nagalingayya M., Mathpati B.S. (2025). Deep Reinforcement Learning-Driven Cooperative Routing for Energy Efficiency in Wireless Multimedia Sensor Networks. Int. J. Commun. Syst..

[13] Rossi E., Chamberlain B., Frasca F., Eynard D., Monti F., Bronstein M. (2020). Temporal Graph Networks for Deep Learning on Dynamic Graphs. arXiv.

[14] Guha S., Khuller S. (1998). Approximation Algorithms for Connected Dominating Sets. Algorithmica.

[15] Wu J., Li H. On Calculating Connected Dominating Set for Efficient Routing in Ad Hoc Wireless Networks. Proceedings of the 3rd International Workshop on Discrete Algorithms and Methods for Mobile Computing and Communications (DIALM).

[16] Wan P.J., Alzoubi K.M., Frieder O. Distributed Construction of Connected Dominating Set in Wireless Ad Hoc Networks. Proceedings of the IEEE INFOCOM.

[17] van Rooij J.M.M., Bodlaender H.L. (2011). Exact Algorithms for Dominating Set. Discret. Appl. Math..

[18] Jiang H., Zheng Z. An Exact Algorithm for the Minimum Dominating Set Problem. Proceedings of the International Joint Conference on Artificial Intelligence (IJCAI).

[19] Wu Z., Pan S., Chen F., Long G., Zhang C., Philip S.Y. (2021). A Comprehensive Survey on Graph Neural Networks. IEEE Trans. Neural Netw. Learn. Syst..

[20] Hamilton W., Ying Z., Leskovec J. Inductive Representation Learning on Large Graphs. Proceedings of the Advances in Neural Information Processing Systems (NeurIPS).

[21] Veličković P., Cucurull G., Casanova A., Romero A., Liò P., Bengio Y. Graph Attention Networks. Proceedings of the International Conference on Learning Representations (ICLR).

[22] Rossi R., Fey A., Leskovec J. (2019). Fast Graph Representation Learning with PyTorch Geometric. arXiv.

[23] Xu D., Ruan C., Korpeoglu E., Kumar S., Achan K. Inductive Representation Learning on Temporal Graphs. Proceedings of the International Conference on Learning Representations (ICLR).

[24] Rossi E., Chamberlain B., Frasca F., Eynard D., Monti F., Bronstein M.M. Temporal Graph Networks. GitHub Repository. https://github.com/twitter-research/tgn.

[25] Aggarwal C., Subbian K. (2014). Evolutionary Network Analysis: A Survey. ACM Comput. Surv..

[26] Kazemi A., Goel M., Neville J. (2022). Representation Learning for Dynamic Graphs: A Survey. J. Artif. Intell. Res..

[27] Ying R., He R., Chen K., Eksombatchai P., Hamilton W.L., Leskovec J. Graph Convolutional Neural Networks for Web-Scale Recommender Systems. Proceedings of the ACM SIGKDD International Conference on Knowledge Discovery and Data Mining.

[28] Cai H., Zheng V.W., Chang K.C.-C. (2018). A Comprehensive Survey of Graph Embedding: Problems, Techniques, and Applications. IEEE Trans. Knowl. Data Eng..

[29] Sankar S., Wu Y., Zhang K. DySAT: Deep Neural Representation Learning on Dynamic Graphs via Self-Attention Networks. Proceedings of the 13th International Conference on Web Search and Data Mining (WSDM).

[30] Prates M.O.R., Avelar P.H.C., Lemos H., Lamb L.C., Vardi M.Y. Learning to Solve NP-Complete Problems: A Graph Neural Network for Decision TSP. Proceedings of the AAAI Conference on Artificial Intelligence.

[31] Kingma D.P., Ba J. Adam: A Method for Stochastic Optimization. Proceedings of the International Conference on Learning Representations (ICLR).

